# Analgesic Effects of Intravenous Ketamine during Spinal Anesthesia in Pregnant Women Undergone Caesarean Section; A Randomized Clinical Trial

**DOI:** 10.5812/aapm.7034

**Published:** 2013-09-01

**Authors:** Shekoufeh Behdad, Mohammad Reza Hajiesmaeili, Hamid Reza Abbasi, Vida Ayatollahi, Zahra Khadiv, Alireza Sedaghat

**Affiliations:** 1 Department of Anesthesiology, Shahid Sadoughi Hospital, Shahid Sadoughi University of Medical Sciences and Health Services, Yazd, Iran; 2 Department of Anesthesiology and Critical Care Medicine, Rasoul Akram Medical Center, Iran University of medical sciences (IUMS), Tehran, Iran; 3 Pain Research Center, Shahid Sadoughi University of Medical Sciences and Health Services, Yazd, Iran

**Keywords:** Anesthesia, Spinal, Cesarean Section, Ketamine, Pain Clinics, Analgesia

## Abstract

**Background:**

Suitable analgesia after cesarean section helps mothers to be more comfortable and increases their mobility and ability to take better care of their infants.

**Objectives:**

Pain relief properties of ketamine prescription were assessed in women with elective cesarean section who underwent spinal anesthesia with low dose intravenous ketamine and midazolam and intravenous midazolam alone.

**Patients and Methods:**

Sixty pregnant women scheduled for spinal anesthesia for cesarean section were randomized into two study groups. Ketamine (30 mg) + midazolam (1 mg = 2CC) or 1mg midazolam (2CC) alone, was given immediately after spinal anesthesia. Pain scores at first, second and third hours after CS operation, analgesic requirement and drug adverse effects were recorded in all patients.

**Results:**

Ketamine group had significant pain relief properties in compare with control group in first hours after cesarean section (0.78 ± 1.09 vs. 1.72 ± 1.22, VAS score, P = 0.00). Total dose of meperidine consumption in women of ketamine group was significantly lower than women of control group (54.17 ± 12.86 vs. 74.44 ± 33.82 mg, P = 0.02). There were no significant drug side effects in participated patients.

**Conclusions:**

Intravenous low-dose ketamine combined with midazolam for sedation during spinal anesthesia for elective Caesarean section provides more effective and long lasting pain relief than control group.

## 1. Background

Suitable analgesia after cesarean section helps mothers to be more comforted and increases their mobility in order to prevent deep vein thrombosis and ability to take better care of their infants (1). Different analgesic drugs had been utilized to block multiple pain pathways and reduce side effects of sedative drugs. Opioids are transferred into the milk and might impact neonates. It seemed that maternal intake of these drugs such as opioids had to be declined (2, 3). Ketamine with sub anesthetic doses has analgesic effects which had been used for chronic pain relief. Several clinical trials have reported that ketamine can be administered during anesthesia to reduce opioids needs for postoperative pain relief. Cochrane review at 2006 reported that “Ketamine in subanesthetic doses is effective in reducing analgesic requirements in the first 24 hours after surgery” (4). Ketamine, also decreases postoperative analgesic consumption due to prevention from opioids tolerance (5). This impact was also seen in using sub anesthetic dosage of ketamine (0.15 mg/kg) during spinal anesthesia for cesarean section (6, 7). We think that a single low dose of ketamine during spinal anesthesia in cesarean section might decline the incidence of breakthrough pain in the first 24 hours postoperatively. 

## 2. Objectives

Present randomized clinical trial was performed for evaluating the impact of sub anesthetic dosage of ketamine on postoperative pain relief and the need for supplemental analgesia in women underwent cesarean section. 

## 3. Patients and Methods

Present double blind clinical trial with parallel design was performed on 60 women who had elective CS in Shahid Sadoughi hospital. Present study was approved in research ethical committee of Shahid Sadoughi University of medical sciences and health services and informs consents were obtained from study participants. Older than 18 years women who had elective cesarean section with ASA (American Society for Anesthesiology) Class I, without history of head trauma, addiction, psychiatric drugs usage, hallucination, delirium, hypertension, intracranial hemorrhagic diseases and ketamine sensitivity were included into the trial. Women with drug sensitivity or any occurrence during CS such as sever hemorrhages that needed blood transfusion and contraindication to spinal anesthesia were excluded. Study participants were selected randomly and with consecutive sampling until reaching to study sample size. Present clinical trial was performed in operation room of Shahid Sadoughi hospital during study period between March and June 2010. Women with computer generated random number table, were randomly allocated into two study groups. Our study intervention was 30 mg ketamine injected for women in ketamine group with 1 mg midazolam in 2 milliner syringes. Women of placebo group received only one milligram midazolam in same two milliliter syringes. Two nurses help researchers for preparing and administering study drugs into the study and control groups in the operation room. Syringes were kept in refrigerator and anesthesia technician in time of CS according to table of computer generated random numbers, allocated syringes into the participated women after spinal anesthesia. With patients in the sitting position, spinal anesthesia was performed by either midline or paramedian approach at the L2-3 or L3-4 level, with a 25G Quinke needle and 1.5 ml of 5% lidocaine. The following drugs were used in the study: lidocaine (orion pharma, UK), midazolam (Aburaihan, Iran) and ketamine (TRITTAU, Germany).

Monitoring included standard anesthesia monitors. The visual analog scale (VAS) scores were taken by a single interviewer and a consistent set of instructions was used throughout the study. The concept of the VAS, which consisted of a 10-cm line with 0 equaling “no pain at all” and 10 being “the worst possible pain” (8). Pain scores, duration between CS and first analgesic prescription (hours) and total analgesic consumption (mg meperidine) during first day after intervention were considered as study outcomes. Pain scores in women were measured at first, second and third hours after cesarean delivery. APGAR scores of neonates were measured at first and fifth minutes after child birth. Our study was double blinded and study participants and researchers were blinded about type of intervention which each woman received. 

### 3.1. Statistical Analysis 

Study data were entered into the SPSS 16.0 software and analyzed with chi-square test for qualitative and student t-test for quantitative variables between trial and control groups. Less than 0.05 calculated p-values were assumed as significant results. 

## 4. Results

In present double blind clinical trial, 60 women were included into data analysis. Mean age and weight in participated women were 28.34 ± 5.15 years and 72.06 ± 15.03 kilogram respectively. Number of pregnancy, delivery and previous CS had no significant differences between two trial groups. Details of basal variables comparison between two trial groups were presented in Table 1. 

**Table 1.** Basal Variables between Study Participants

Variable	Ketamine (n = 30), Mean ± SD	Midazolam (n = 30), Mean ± SD	P value
**Age, y, Mean ± SD**	27.4 ± 4.80	29.31 ± 5.41	0.16^a^
**Weight, Kg**	73.07 ± 10.77	70.29 ± 1.07	0.60^a^
**Gravity**	2 ± 0.93	2.29 ± 1.07	0.29^a^
**Parity**	1.89 ± 0.85	2.04 ± 1.04	0.57^a^
**Number of previous CS**	1.13 ± 0.64	1.14 ± 0.73	0.97^b^
**Systolic blood pressure, mmhg**	131.20 ± 12.84	131.30 ± 13.77	0.97^a^
**Diastolic blood pressure, mmhg**	84.27 ± 11.39	82.23 ± 11.83	0.50^a^
**Heart rate, Bit/minute**	96.97 ± 11.62	94.53 ± 16.25	058^a^

^a^calculated with independent sample t-test

^b^calculated with Chi-square test

Mean of pain scores in first hours after CS operation, in ketamine group (0.78 ± 1.09) was significantly lower than control group (1.72 ± 1.22; P = 0.00). Mean of postoperative duration until prescribing first analgesic drug in women of ketamine group (5.63 ± 2.60 hours) was significantly longer than women of control group (4.18 ± 2.05 hours; P = 0.03). Women in ketamine group received significantly lower meperidine compared to women of control group (54.17 ± 12.86 vs. 74.44 ± 33.82 mg, P = 0.02) (Table 2). 

**Table 2.** Pain Scores and Analgesic Consumption in Women after Trial Intervention Between Two Groups

Variable	Ketamine (n = 30), Mean ± SD	Midazolam (n = 30), Mean ± SD	P value^a^
**Pain scores in the first hours after CS (VAS score)**	0.78 ± 1.09	1.72 ± 1.22	0.00
**Pain scores in the second hours after CS (VAS score)**	3.44 ± 1.76	3.41 ± 1.64	0.95
**Pain scores in the third hours after CS (VAS score)**	4.62 ± 1.42	4.97 ± 1.12	0.31
**Duration between CS and first analgesic prescription, h**	5.63 ± 2.60	4.18 ± 2.05	0.03
**Total analgesic consumption (Mg ** ***Meperidine*** **) **	54.17 ± 12.86	74.44 ± 33.82	0.02

^a^ calculated with independent sample t-test

Comparison of side effects of study drugs in women after trial intervention between two is groups presented in Table 3. 

**Table 3.** Comparison of Side Effects of Study Drugs in Women after Trial Intervention Between Two Groups

Variable	Ketamine (n = 30), Mean ± SD	Control (n = 30), Mean ± SD	P value^a^
**Post-operative vomiting**	13.3	10	> 0.05
**Hallucination**	6.66	3.33	> 0.05
**Recovery stay**	42.98 ± 12.98	42.77 ± 5.79	0.29
**APGAR Score (First minute)**	8.9 ± 0.31	8.87 ± 0.57	0.78
**APGAR Score (Fifth minute)**	9.93 ± 0.25	9.97 ± 0.18	0.56
**Umbilical blood PH**	7.27 ± 0.02	7.28 ± 0.02	0.23

^a^P value calculate with chi-square and independent sample t-test

## 5. Discussion

Present double blinded control trial evaluated effects of prescribing ketamine (30 mg) with spinal anesthesia in 60 women undergone elective cesarean section. Ketamine had significant pain relief properties in comparison with placebo in the first hours after CS operation. Total analgesic consumption in women of ketamine group was significantly lower than women of control group. Role of ketamine in reducing need for opium analgesia was reported in several studies (9-12). Researchers used ketamine as intravenous patients control analgesia (IVPCA) (9-12), IV continues (13-15), IV continues with epidural opium (16-18), IV ketamine with IV opium (19-21), ketamine epidural (17, 21, 22) and topical ketamine in pediatric analgesia for tonsillectomy (23, 24). In gynecology surgery, there were some studies which had been used ketamine in subanesthetic dosage. Sen et al. reported that women who received ketamine (0.15 mg/kg) during spinal anesthesia for CS operation had declined diclofenac recruitment in the first day postoperatively (7). Kwol et al. in laparoscopic gynecology surgery reported that reduced requirement to paracetamol in the first week postoperatively in women who received ketamine (0.15 mg/kg) (25). Against above studies, Dahl et al. reported no opioids-sparing effects with single doses of ketamine (0.4 mg/kg) in women who underwent abdominal hysterectomy (19). In non-gynecology operation, single doses of ketamine (0.05-0.15 mg/kg) reduced opioids consumption following orthopedic surgery with sustained effects up to three days after arthroscopy (19, 26). Clinical trials which used low-dose ketamine had been suggested a number of mechanisms for the analgesic effects. These include supraspinal effects, prevention of acute opioid tolerance and suppression of central sensitization, a phenomenon by which dorsal root neurons increase their spontaneous discharge rate, responsiveness and enlarge their receptive field in response to repeated painful stimulus (4). Cesarean delivery can lead to chronic pain in 6–8% of patients and based on our results, future studies investigating melamine’s role in reducing chronic pain after cesarean delivery are warranted (27, 28).

Women in our study had no considerable side effects and ketamine was tolerated well. Ketamine had some side effects such as itching, urine suppression, hallucination, nausea and vomiting in women and some adverse impacts on neonatal parameters such as Apgar score in first and fifth minutes but PH of umbilical blood did not have significant differences with placebo groups (29). Similar to our study, Meer et al. reported that ketamine in anesthesia for cesarean section had lower side effects (30). Lack of checking patients and surgeon satisfaction is one of the drawbacks of our study. It is recommended that due to significant postoperative pain relief without considerable side effects, ketamine in low dosage (less than 1 mg/kg) might be useful in women undergoing elective cesarean section for decline their need to other analgesic drugs. 
